# Correcting for the heterogeneous boron distribution in a tumor for BNCT dose calculation

**DOI:** 10.1038/s41598-023-42284-x

**Published:** 2023-09-21

**Authors:** Yi-Chiao Teng, Jiang Chen, Wan-Bing Zhong, Yuan-Hao Liu

**Affiliations:** 1 Neuboron Therapy System Ltd., Xiamen, Fujian Province People’s Republic of China; 2 National Tsing Hua University, Hsinchu, 30013 Taiwan Republic of China; 3 Nanjing Vocational University of Industry Technology, Nanjing, Jiangsu Province People’s Republic of China; 4 Nanjing University of Aeronautics and Astronautics, Nanjing, Jiangsu Province People’s Republic of China; 5 Neuboron Medtech Ltd., Nanjing, Jiangsu Province People’s Republic of China; 6 Xiamen Humanity Hospital, Xiamen, Fujian Province People’s Republic of China

**Keywords:** Medical research, Oncology, Engineering, Mathematics and computing, Physics

## Abstract

Most treatment planning systems of boron neutron capture therapy perform dose calculations based on the assumption of a homogeneous boron distribution in tumors, which leads to dose distortion due to the difference between the tumor-to-normal tissue ratio (TNR) range measured in positron emission tomography images (PET) and the target delineation in computed tomography images of the treatment plan. The heterogeneous boron distribution in the target of the treatment plan can be obtained by image fusion. This study provides a way to quantify a heterogeneous boron distribution based on PET images. Theoretically, the same mean TNR for dose calculation by homogeneous or heterogeneous boron distribution should get almost the same mean dose. However, slightly different mean doses are found due to the partial volume effect for a small target volume. The wider the boron distribution is, the higher the impact on the dose-volume histogram distribution is. Dose distribution with homogeneous boron distribution may be overestimated in low boron uptake regions by wrong boron concentration and neutron flux depression. To accurately give the tumor prescription dose and achieve better tumor control, for low dose regions of the tumor should be considered more boron neutron capture therapy treatments or combined with other treatment modalities. The heterogeneous boron distribution must be taken into consideration to have an accurate dose estimation. Therefore, the way how medical physicists and clinicians process the TNR in gross tumor volume should be refined, and the method demonstrated in the work provides a good reference.

## Introduction

The boron neutron capture therapy (BNCT) dose consists of the physical boron-10 dose (D_phy,B10_, unit: Gy), physical neutron dose (D_phy,N_, unit: Gy) from ^1^H(n,n')p and ^14^N(n,p) ^14^C, and physical photon dose (D_phy,P_, unit: Gy) which includes primary photon from the source port and secondary photon dose from neutrons reacting in the human body elements. Considering the relative biological effectiveness (RBE)^1^ of different radiation types and compound biological effectiveness (CBE)^2^ of different boron-containing drugs to the target tissue. The BNCT bioequivalent dose (D_BNCT_, unit: Gy-Eq) calculation formula is shown in Eq. (1)^3^ and the following values are used in this study: RBE = 3.2 for neutron (RBE_N_), RBE = 1.0 for photon (RBE_P_), and CBE = 3.8 for tumor^4^.

1 $$\begin{array}{c}{D}_{BNCT}={D}_{phy,B10}\cdot CBE+{D}_{phy,N}\cdot {RBE}_{N}+{D}_{phy,P}\cdot {RBE}_{P}\end{array}$$

The main dose contribution for BNCT is the D_phy,B10_ generated by the ^10^B(n,α) ^7^Li, boron neutron capture reaction^5^. Lesions that could be effectively treated with BNCT are areas of significant accumulation of boron-containing drugs, such as boronophenylalanine (BPA), which is now the most widely applied BNCT drug clinically^6^; the exact quantity of the boron-containing drug that enters the tumor area during treatment directly affects the quantity of delivered dose. In recent years, BNCT has moved toward a theragnostic approach to ensure that the distribution of boron-containing drugs in the target region of patients can be determined in advance by using diagnostic drugs or equipment^7, 8^; for instance, the BPA distribution in a tumor can be obtained through PET scan images of^18^F-BPA^8–10^. The mean standardized uptake value (SUV_mean_) ratio of tumor and normal tissue derived from PET images represents the BPA concentration ratio, namely, the TNR which is shown in Eq. (2)^11^. However, BPA accumulation correlates with tumor cell activity. In clinical trials, a TNR higher than 2.5 is a widely adopted criterion for BNCT^12, 13^. Boron concentration as well as its following induced boron dose is the major contributor to the total absorbed dose of region of interest (ROI), especially in tumors. Therefore, quantifying the boron distribution is crucial for optimizing a treatment plan and accurately assessing the dose distribution in a tumor.

2 $$\begin{array}{c}TNR=\frac{{SUV}_{mean,\; tumor}}{{SUV}_{mean, normal\; tissue}}\end{array}$$

In clinical applications, the heterogeneous BPA concentration in a tumor should be determined from ^18^F-BPA PET images to ensure the BNCT dose is correctly evaluated^14^. The contemporary treatment planning systems (TPSs) employed are delineated in Table 1^15–19^. These systems possess the capability to interpret DICOM (Digital Imaging and Communications in Medicine) images, encompassing modalities such as CT (Computed Tomography), MRI (Magnetic Resonance Imaging), PET, or the RT (Radiotherapy) structure set. Notably, none of the TPSs currently tailored for BNCT have harnessed PET images for the estimation of boron concentration distribution. Instead, a given, fixed TNR value has been universally implemented, signifying a uniform boron distribution within the ROI. This methodological approach could potentially compromise the precision of dose evaluation.

**Table 1:** Treatment planning systems for BNCT.

TPS	Developers	Boron distribution in ROI	Dose engine
SERA	INEEL/MSU	Homogeneous	seraMC
Tsukuba Plan	Univ. of Tsukuba	Homogeneous	PHITS
NeuCure	RaySearch/ SHI	Homogeneous	PHITS
NeuMANTA	Neuboron	Homogeneous/ Heterogeneous	COMPASS
TH-BNCTplan	NTHU	Homogeneous	MCNP

INEEL: Idaho National Engineering and Environmental Laboratory; MSU: Montana State University; SHI: Sumitomo Heavy Industries; NTHU: National Tsing Hua University.

Researchers have attempted to introduce PET images into a TPS named BDTPS (Boron Distribution Treatment Plan System)^20^ to calculate the boron dose; however, BDTPS uses the Snyder phantom model instead of patient CT images for the dose calculation, which does not correctly reproduce the true geometry and location of a tumor in a patient; such that BDTPS has eventually not been used in clinical application. Nichols et al. attempted to import boron distribution information from PET images into SERA (Simulation Environment for Radiotherapy Applications)^21^ for dose calculation but did not describe the calculation method used or the difference in the calculated results before and after the application of the PET images; hence, this approach has not been used in clinical application either. NeuMANTA (Multifunctional Arithmetic for Neutron Transportation Analysis) is a new generation BNCT-specific TPS with dose calculation engine, COMPASS (COMpact PArticle Simulation System)^22^, developed by Neuboron Medical Group. To increase the accuracy of the boron dose calculation, a three-dimensional boron concentration distribution model based on PET images has been developed in NeuMANTA with the aid of a related calculation module and image module, thereby realizing feasibility for clinical application for the first time.

In BNCT, the spatial distribution of the boron dose within the tumor is primarily determined by the product of the spatial distribution of boron-10 atoms and the spatial distribution of thermal neutrons. Specifically, boron-10 atoms, possessing a relatively high neutron capture cross-section, exert a direct influence on the spatial distribution of thermal neutrons, giving rise to a self-shielding effect^23^. Consequently, the spatial distribution of boron-10 atoms both directly and indirectly impacts the boron dose distribution within the tumor.

This study initiates an innovative approach by utilizing the SUV values, derived from PET images, to ascertain the spatial distribution of boron-containing drugs within the tumor. This spatial information is subsequently applied in TPSs for dose calculation. This proposed method enhances the precision of boron dose distribution characterization in BNCT. Additionally, the research provides an examination of the differences in spatial dose distribution and the Dose-Volume Histogram (DVH), offering a comprehensive comparison between the new technique introduced and established conventional practices.

## Methods

The normalized radioactivity is determined by calculating the SUV from^18^F-BPA PET images, which is defined as the ratio of the tracer radioactivity uptake by the local tissue to the average injection of radioactivity into the whole body. Therefore, the SUV can reflect the ^18^F-BPA uptake of the ROI. The SUV of the ROI can be determined using the Eq.(3)^24, 25^:

3 $$\begin{array}{c}SU{V}_{\text{body\; weight}}\left(\text{kg/ml}\right)=\frac{A\text{citivity Concentration in ROI}\left(\text{Bq/ml}\right)}{\left(\frac{Injected\; Dose\left(Bq\right)}{body \;weight\left(kg\right)}\right)}\end{array}$$

In this study, the SUV of a voxel is obtained from the voxel finite element intensity in the PET images with attenuation corrected, which in turn is used to determine the ^18^F-BPA distribution in the ROI. In the calculation, the degree of decay of the tracer radioactivity from the measurement time to the scan time is considered, and the decay of the tracer during imaging is neglected during imaging, resulting in the Eq. 4 for determining the SUV from the voxel intensity in the PET images^26^:

4 $$\begin{array}{c}SU{V}_{voxel}=\frac{PET \;\text{image voxel intensity}\times Attenuation\; Correction \;Factor \, \times Body \;weight}{Injected \;Dose\times {2}^{-\frac{\text{Measuremet time-Scan time}}{H\text{alf time}}}}\end{array}$$

The TNR defined as the ratio of the mean SUV (SUV_mean_) between tumor and normal tissue, as shown in Eq. 2. The baseline voxel intensity for SUV_mean,normal tissue_ and SUV_mean,tumor_ can be determined using Eq. 3. The heterogeneous boron distribution in the tumor is determined by dividing the SUV of each tumor ROI voxel by SUV_mean,normal tissue_. Note that the tumor ROI region is defined by using CT images rather than using PET images alone.

The heterogeneous boron distribution is accounted for by grouping the boron concentration of each voxel in the ROI into *I* groups. The groupwise is performed using the Eq. 5:

5 $$\begin{array}{c}{\overline{SUV} }_{ROI,i}=\frac{{SUV}_{ROI,upper}-{SUV}_{ROI,lower}}{I}\times \left(i-0.5\right)+{SUV}_{ROI,lower} i=1, 2, 3, 4, \dots , I\end{array}$$

where *I* is the total number of groups used in the groupwise, based on an even divide; and SUV_ROI,upper_ and SUV_ROI,lower_ are the upper and lower limits of the SUVs of the ROI, respectively.

However, groupwise may lead to a slight change in the total number of boron atoms in the ROI. Therefore, a normalization factor $k$ (as defined in Eq. 6) is applied to ensure that the total number of boron atoms remains the same after groupwise (i.e., Eq. 7):

6 $$\begin{array}{c}k=\frac{{\int }_{V}{SUV}_{ROI}\left(V\right)dV}{\sum_{i}{N}_{i}{\overline{SUV} }_{ROI,i}}\end{array}$$

7 $$\begin{array}{c}{\int }_{V}{SUV}_{ROI}\left(V\right)dV=k\sum_{i}{N}_{i}{\overline{SUV} }_{ROI,i}\end{array}$$

where *N*_*i*_ stands for the number of counts of the ith group, and SUV_ROI_(V) represents the SUV of a voxel located at the V position (or the Vth voxel in the ROI).

To assign the boron concentration of each material in the ROI, the number of boron atoms N_B10,group_(V) in the respective material is determined by using the Eq. 8:

8 $$\begin{array}{c}{N}_{B10,group}\left(V\right)=\xi \times k\times {\overline{SUV} }_{ROI,i}\end{array}$$

where the group index *i* is determined as the Eq. 9:

9 $$\begin{array}{c}i=\lceil\frac{{I\times (SUV}_{ROI}(V)-{SUV}_{ROI,lower})}{{SUV}_{ROI,upper}-{SUV}_{ROI,lower}}\rceil\end{array}$$

where $\xi$ is the conversion factor between the number of boron atoms and the SUV. If the ROI contains M kinds of materials, the total number of materials M_total_ used in the dose calculation for the ROI is following the Eq. 10:

10 $$\begin{array}{c}{M}_{total}=M\times I\end{array}$$

The Eq. 10 clearly shows that it is neither necessary nor practical to use a large number (e.g., 100 or higher) for *I*. A very large M_total_ will consume considerable computer memory and computation power. The use of *k* ensures that the neutron suppression in the ROI is not significantly affected by using a relatively small*I*(50, for instance). However, further investigation is needed to determine the optimal *I* for individual cases.

Upon defining the N_B10,group_ and specifying the material information for each voxel within the ROI, one can determine the number of capture reactions corresponding to each boron-10 atom. This quantity is symbolized as R_B10_ and has units of capture reactions per boron-10 atom. This determination is calculated by the Monte Carlo dose simulation engine. The energy liberated during each boron-10 capture reaction is denoted as E_cap_, measured in joules per capture reaction. A prevailing assumption is that the energy derived from the produced ^4^He and ^7^Li ions remains localized and is directly deposited at its inception point.

The physical dose due to boron-10, represented as D_phy,B10_, can be computed utilizing Eq. (11) for a particular voxel at position V, Mass(V) and N_B10_(V) (expressed in kilograms per voxel and boron-10 atoms per voxel, respectively) depict the mass and the total number of boron-10 atoms:

11 $$\begin{array}{c}{D}_{phy,B10}\left(V\right)=\frac{{N}_{B10}\left(V\right)\times {R}_{B10}\times {E}_{cap}}{Mass\left(V\right)}\end{array}$$

The relationship for N_B10_(V) is given by:

12 $$\begin{array}{c}{N}_{B10}\left(V\right)={\xi \times SUV}_{ROI}\left(V\right)\end{array}$$

Here, we use the original SUV of the Vth voxel, which is closer to the real boron dose than the groupwise value. Note that the variable V can be represented by using the corresponding x, y, and z indexes in the voxel model to indicate the location of V in space. To prevent the use of an excessively large number of types of materials, NeuMANTA divides the voxel boron concentration ratio into 50 different boron concentrations (groupwise) and assigns these concentrations to the corresponding tumor material to calculate the boron dose.

**Figure 1:**
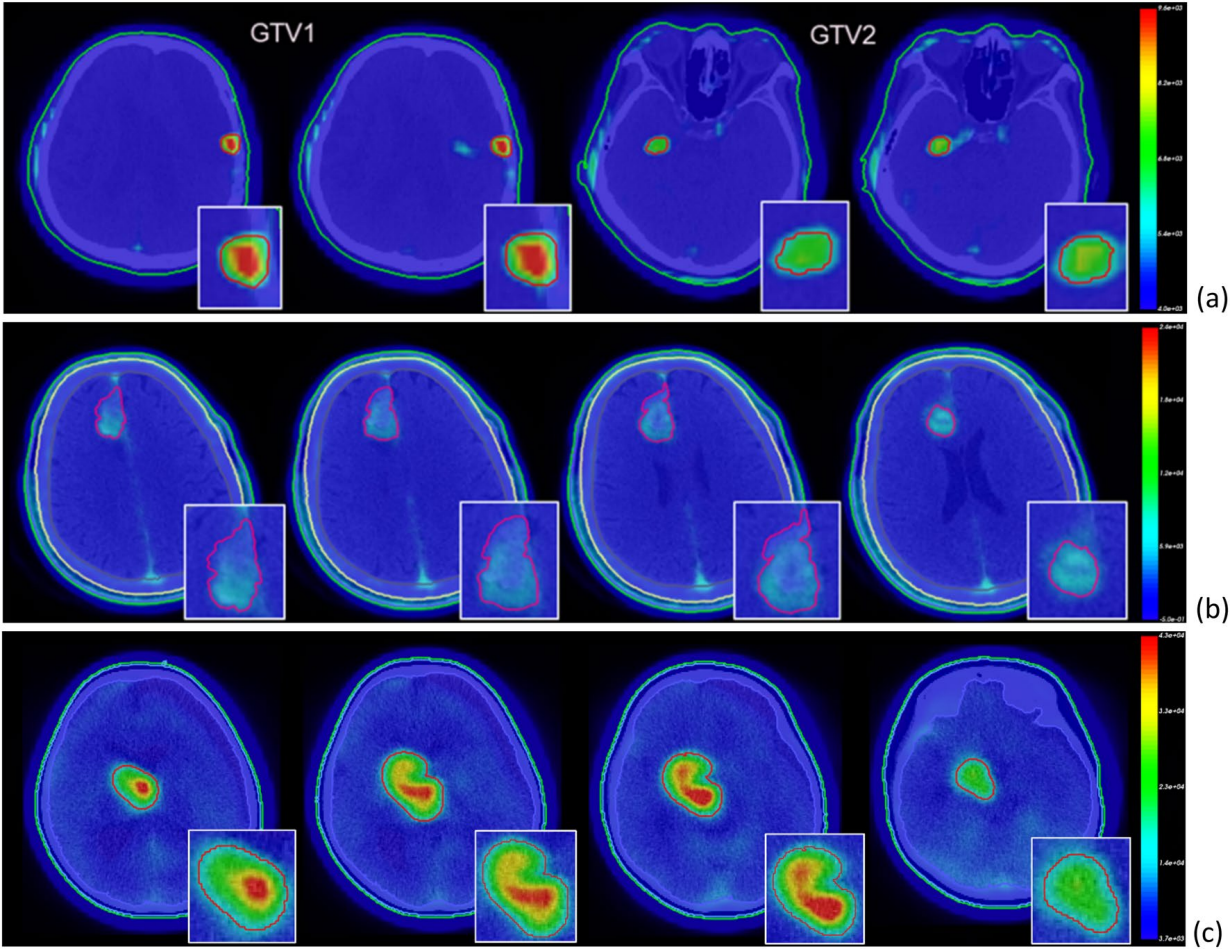
^18^F-BPA distribution of PET/CT fused images for (**a**) Case 1, (**b**) Case 2 and (**c**) Case 3.

Table 2 is a PET/CT image information of real glioblastoma (GBM) cases. These cases are used to compare the dose results obtained using an assumed homogeneous BPA distribution (homo-boron method) and the quantified heterogeneous BPA distribution (hetero-boron method) based on ^18^F-BPA PET/CT images in the tumor region. The PET/CT image fusion technique of NeuMANTA is applied to determine the boron distribution in the gross tumor volume (GTV), as shown in Fig. 1. Figure 1a shows Case 1 of 67-year-old male has a small tumor GTV1 in the left brain lobe with a significantly inhomogeneous ^18^F-BPA distribution and a small tumor GTV2 in the right brain lobe with a more uniform drug distribution. Figure 1b shows Case 2 of 59-year-old male has a tumor GTV in the frontal lobe, for which ^18^F-BPA has accumulated in only part of the volume. Figure 1c shows Case 3 of 8-year-old male has a brain stem tumor with high drug accumulation.

**Table 2:** PET/CT image information of real brain tumor cases.

	CT image		PET image		Manufacturer, model	Provider
	Matrix	Voxel (mm^3^)	Matrix	Voxel (mm^3^)		
Case 1	512 × 512	0.98 × 0.98 × 3.75	128 × 128	5.47 × 5.47 × 3.27	GE, Discovery STE	VGHTPE
Case 2	512 × 512	0.68 × 0.68 × 1.87	512 × 512	1.18 × 1.18 × 1.87	Siemens, Biograph 64	PUMCH
Case 3	512 × 512	0.98 × 0.98 × 3.75	128 × 128	3.91 × 3.91 × 3.27	GE, Discovery STE	VGHTPE

VGHTPE: Taipei Veterans General Hospital; PUMCH: Peking Union Medical College Hospital.

## Results

**Figure 2:**
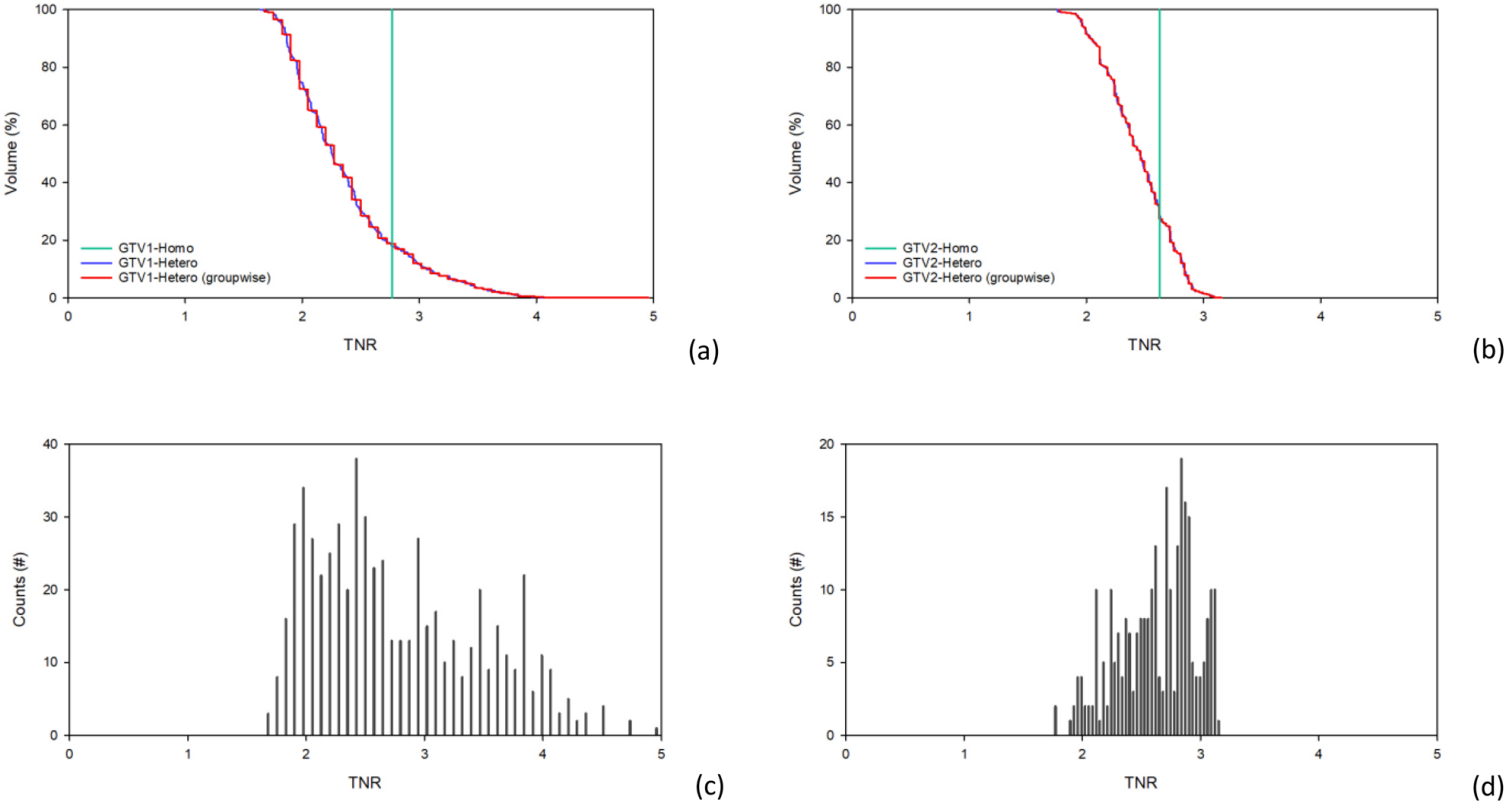
(**a**) TNR-volume histogram for GTV1, (**b**) TNR-volume histogram for GTV2, (**c**) TNR-counts bar chart for GTV1, and (**d**) TNR-counts bar chart for GTV2 for Case 1.

The Eq. 4 is used to determine the baseline voxel intensity of the brain in Case 1. GTV1 is defined based on the CT images, the calculated TNR range is from 1.64 to 4.92 with an average of 2.77, and the GTV2 TNR range is from 1.75 to 3.14 with an average of 2.62. The TNR-volume histogram and TNR-counts bar chart are shown in Fig. 2. Table 3 shows the dose rates calculated using both the homo-boron and hetero-boron methods, and the difference between the results for Case 1. The calculation errors in the maximum dose rate (${\dot{D}}_{max}$) are less than 0.72%. The difference between the minimum dose rates (${\dot{D}}_{min}$) in GTV1 and GTV2 calculated using the homo-boron and hetero-boron methods is 19.1% and 26.1%, respectively. As the same total neutron flux in the tumor area is determined using the homo-boron and hetero-boron methods, the mean dose rates (${\dot{D}}_{mean}$) of the tumor determined using the two methods should be close. The difference between the ${\dot{D}}_{mean}$ obtained using the two methods for GTV1 and GTV2 is 2.3% and 2.0%, respectively. This result is mainly due to the partial volume effect^27, 28^ of the large difference in the voxel size between the PET and CT images. In addition, the small tumor volume produces a significant partial volume effect. The ${\dot{D}}_{80}$ is the dose rate for covering 80% of the GTV volume and often used as a prescription dosage index. The difference between the ${\dot{D}}_{80}$ calculated by the homo-boron and hetero-boron methods for GTV1 and GTV2 is 12.3% and 9.2%, respectively.

**Table 3:** Dose rate results for Case 1 obtained using the homo-boron and hetero-boron methods.

ROI	Method	${\dot{D}}_{max}$(cGy-Eq/s)	${\dot{D}}_{min}$(cGy-Eq/s)	${\dot{D}}_{mean}$(cGy-Eq/s)	${\dot{D}}_{80}$(cGy-Eq/s)
GTV1	Homo-boron	1.908	1.016	1.480	1.283
	Hetero-boron	2.344	0.822	1.446	1.125
	Difference	22.9%	− 19.1%	− 2.3%	− 12.3%
GTV2	Homo-boron	1.899	1.712	1.808	1.770
	Hetero-boron	2.060	1.266	1.772	1.607
	Difference	8.5%	− 26.1%	− 2.0%	− 9.2%

**Figure 3:**
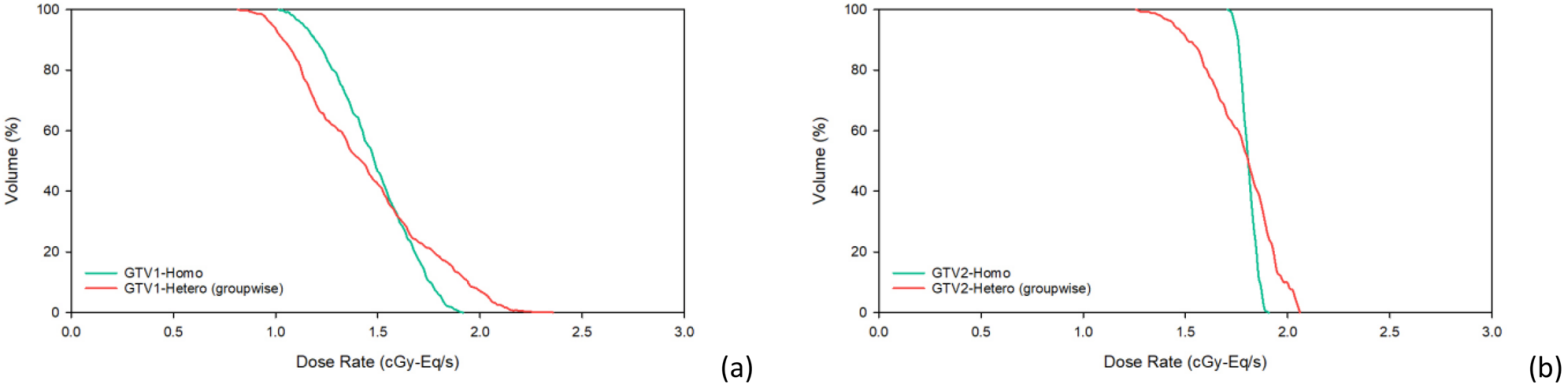
(**a**) DVH of GTV1, (**b**) DVH of GTV2 with homo-boron and hetero-boron methods for Case 1.

Table 4 shows dose components (the boron dose rate, ${\dot{D}}_{B}$; the neutron dose rate, ${\dot{D}}_{N}$; and the photon dose rate, ${\dot{D}}_{P}$) at ${\dot{D}}_{max}$ and ${\dot{D}}_{min}$ in the tumor, where the dose percentage is shown in parentheses. The TNR of GTV1 ${\dot{D}}_{min}$ calculated by the hetero-boron method is 1.91, and the corresponding ${\dot{D}}_{B}$ is 0.561 cGy-Eq/s. There is a difference of 31.0% for the TNR and of 25.6% for ${\dot{D}}_{B}$ between the results obtained using the hetero-boron and homo-boron methods. The TNR of GTV2 ${\dot{D}}_{min}$ calculated using the hetero-boron method is 1.96, and the corresponding ${\dot{D}}_{B}$ is 1.062 cGy-Eq/s. There is a difference of 25.1% for the TNR and of 29.8% for ${\dot{D}}_{B}$ between the results obtained using the two methods. Figure 3 shows the DVH of the tumor for Case 1.

**Table 4:** Components of the dose rate at the ${\dot{D}}_{max}$ and ${\dot{D}}_{min}$ dose category in the tumor for Case 1 calculated using the homo-boron and hetero-boron methods.

Dose category	Method	TNR	${\dot{D}}_{B}$(cGy-Eq/s)	${\dot{D}}_{N}$(cGy-Eq/s)	${\dot{D}}_{P}$(cGy-Eq/s)
GTV1 ${\dot{D}}_{max}$	Homo-boron	2.77	1.669 (87.5%)	0.096 (5.0%)	0.142 (7.4%)
	Hetero-boron	4.18	2.095 (89.4%)	0.131 (5.6%)	0.118 (5.0%)
	Difference	50.9%	25.5%	36.5%	− 16.9%
GTV1 ${\dot{D}}_{min}$	Homo-boron	2.77	0.754 (74.2%)	0.166 (16.3%)	0.096 (9.4%)
	Hetero-boron	1.91	0.561 (68.2%)	0.166 (20.2%)	0.096 (11.7%)
	Difference	− 31.0%	− 25.6%	0.0%	0.0%
GTV2 ${\dot{D}}_{max}$	Homo-boron	2.62	1.677 (88.3%)	0.067 (3.5%)	0.155 (8.2%)
	Hetero-boron	2.53	1.851 (89.9%)	0.049 (2.4%)	0.158 (7.7%)
	Difference	− 3.4%	10.4%	− 26.9%	1.9%
GTV2 ${\dot{D}}_{min}$	Homo-boron	2.62	1.514 (88.4%)	0.048 (2.8%)	0.151 (8.8%)
	Hetero-boron	1.96	1.062 (83.9%)	0.051 (4.0%)	0.153 (12.1%)
	Difference	− 25.1%	-29.8%	5.2%	1.7%

**Figure 4:**
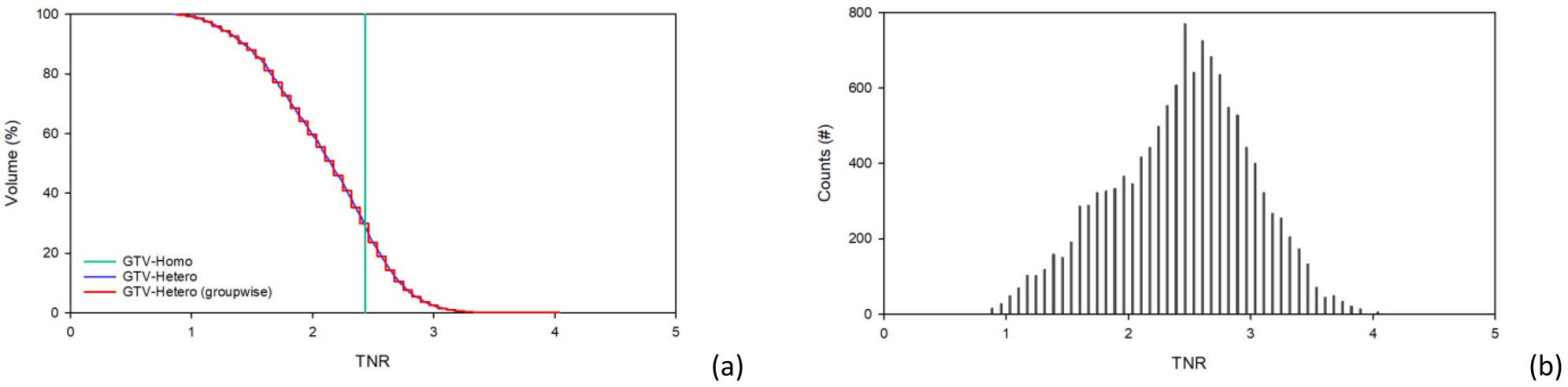
(**a**)TNR-volume histogram, (**b**) TNR-counts bar chart for GTV for Case 2.

**Figure 5:**
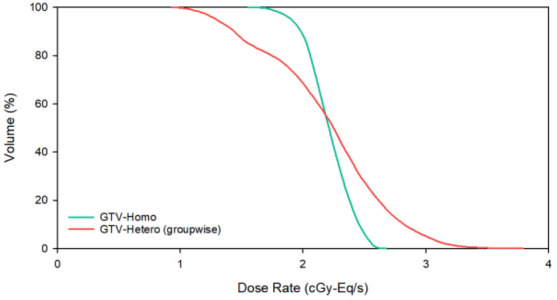
DVH of GTV with homo-boron and hetero-boron methods for Case 2.

In Case 2, the TNR is 2.56 based on the threshold set for the tumor area in the PET image, which is smaller than the GTV delineated by the CT images. Therefore, calculating the uniform boron distribution dose in the GTV by defining the TNR based on the threshold of the PET images could severely distort the drug dose in the GTV. The TNR of the GTV calculated using the quantified non-uniform drug distribution method ranges from 0.85 to 4.00 with an average value of 2.43. Figure 4 shows the TNR-volume histogram and distribution of the TNR counts. There is a 0.7% difference between the ${\dot{D}}_{mean}$ in the GTV calculated using the homo-boron and hetero-boron methods, which is consistent with the total neutron flux in the tumor area being theoretically almost the same for both methods. The GTV bioequivalent dose rate results are shown in Table 5. The ${\dot{D}}_{max}$ and the ${\dot{D}}_{min}$ components are shown in Table 6. The homo-boron method severely overestimates GTV ${\dot{D}}_{min}$ by 40.1%, and the ${\dot{D}}_{80}$ is overestimated by 15.2%. Figure 5 shows the DVH, where the green line is the result obtained using the homo-boron method, and the GTV dose rate ranges from 2.208 to 2.658 cGy-Eq/s. The red line is the result obtained using the hetero-boron method, where the non-uniform boron concentration is accounted for to make the dose distribution range from 0.935 to 3.767 cGy-Eq/s.

**Table 5:** Dose rate results for Case 2 obtained using the homo-boron and hetero-boron methods.

ROI	Method	${\dot{D}}_{max}$(cGy-Eq/s)	${\dot{D}}_{min}$(cGy-Eq/s)	${\dot{D}}_{mean}$(cGy-Eq/s)	${\dot{D}}_{80}$(cGy-Eq/s)
GTV	Homo-boron	2.658	1.560	2.208	2.056
	Hetero-boron	3.767	0.935	2.193	1.744
	Difference	41.7%	− 40.1%	− 0.7%	− 15.2%

**Table 6:** Components of the dose rate at the ${\dot{D}}_{max}$ and ${\dot{D}}_{min}$ dose category of the tumor in Case 2 obtained using the homo-boron and hetero-boron methods.

Dose Category	Method	TNR	${\dot{D}}_{B}$(cGy-Eq/s)	${\dot{D}}_{N}$(cGy-Eq/s)	${\dot{D}}_{P}$(cGy-Eq/s)
GTV ${\dot{D}}_{max}$	Homo-boron	2.43	2.296 (86.4%)	0.136 (5.1%)	0.226 (8.5%)
	Hetero-boron	3.82	3.414 (90.7%)	0.126 (3.3%)	0.226 (6.0%)
	Difference	57.2%	48.7%	− 7.4%	0.0%
GTV ${\dot{D}}_{min}$	Homo-boron	2.43	1.315 (84.3%)	0.105 (6.7%)	0.140 (9.0%)
	Hetero-boron	1.12	0.644 (68.9%)	0.124 (13.3%)	0.167 (17.9%)
	Difference	− 53.9%	− 51.0%	18.1%	19.3%

**Figure 6:**
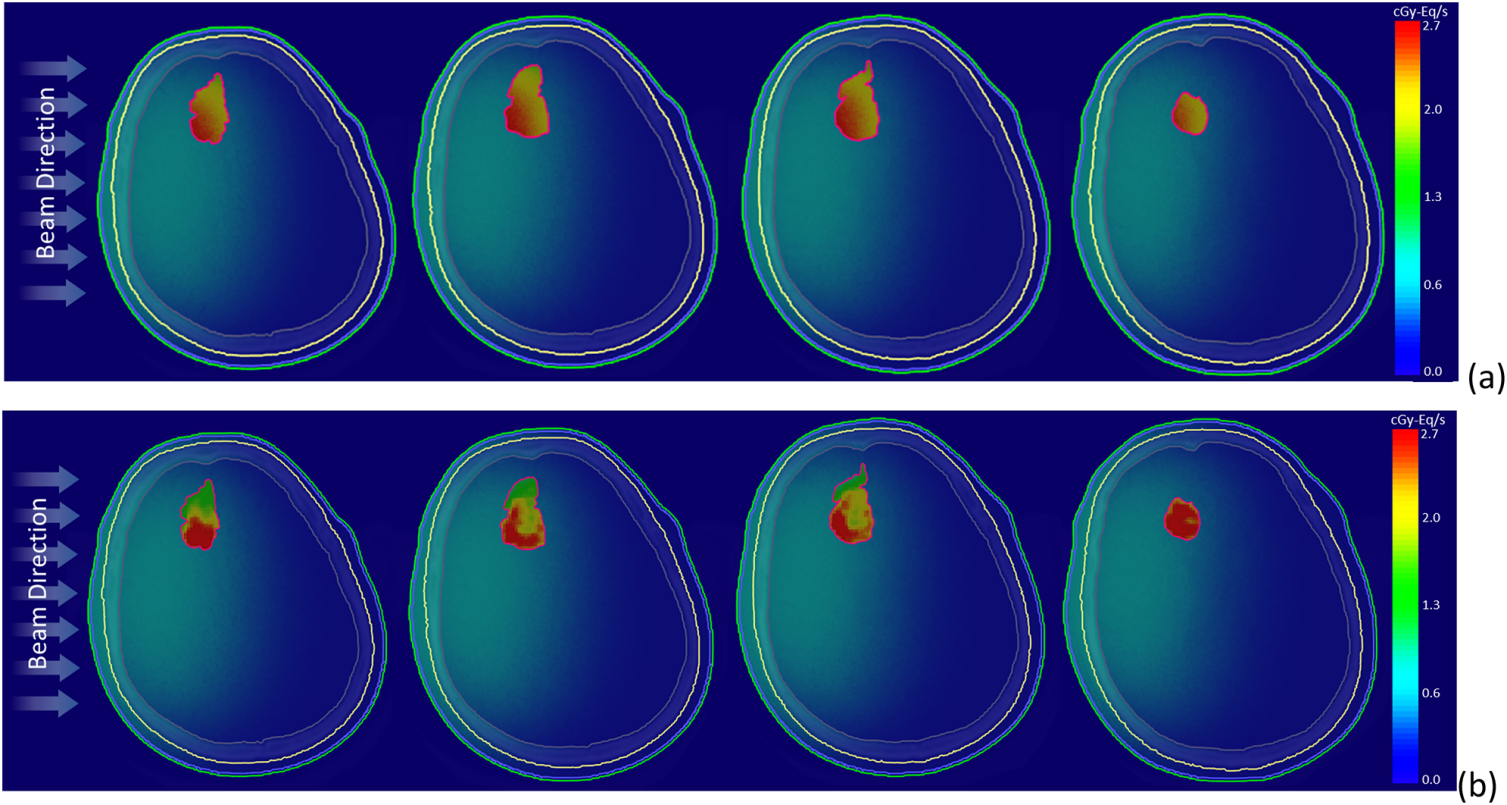
Bioequivalent dose maps of (**a**) homo-boron method, and (**b**) hetero-boron method for Case 2.

**Figure 7:**
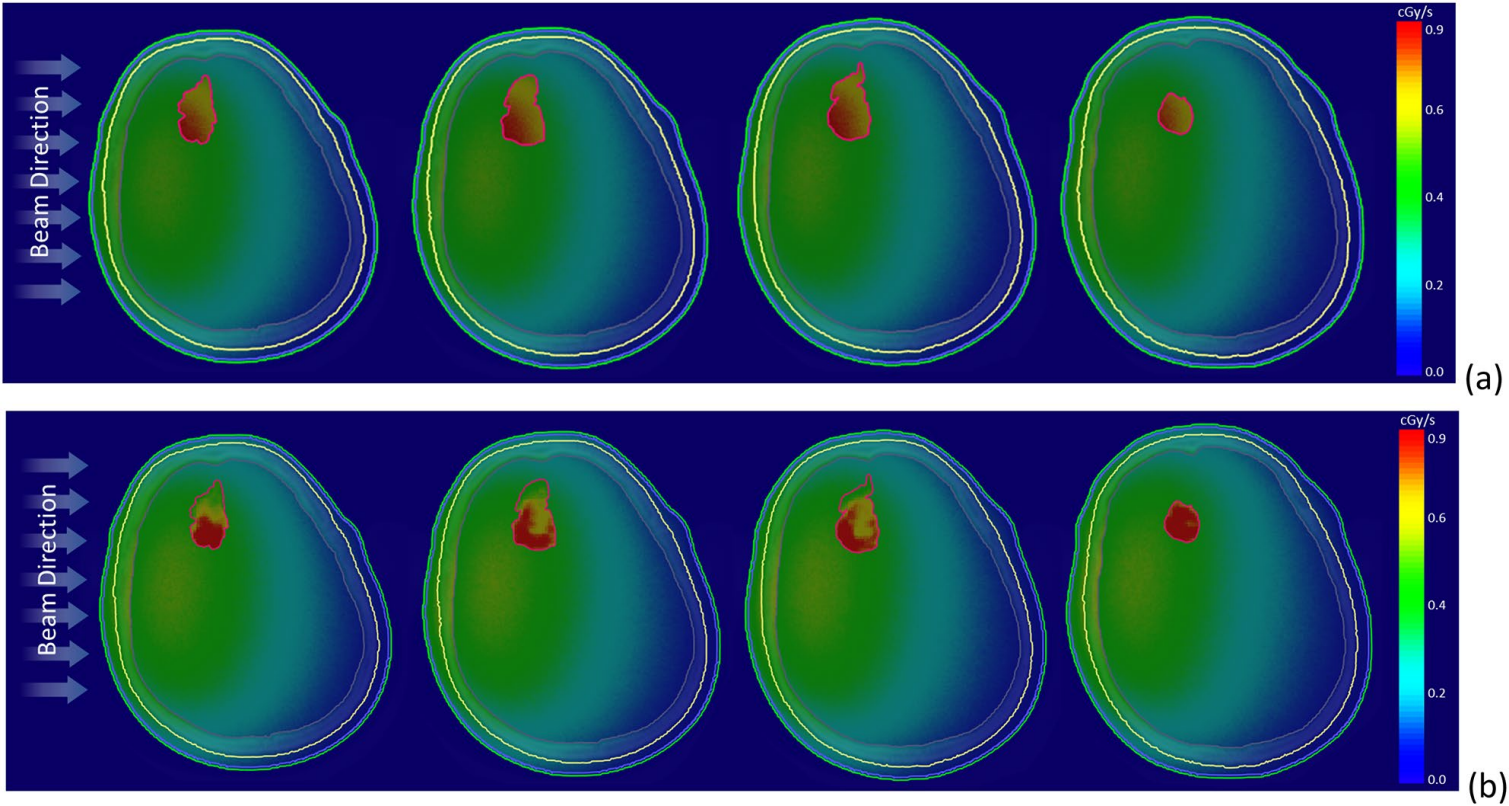
Physical dose maps of (**a**) homo-boron method, and (**b**) hetero-boron method for Case 2.

Figure 6 illustrates the two-dimensional bioequivalent dose rate map, employing both homo-boron and hetero-boron methods. In Fig. 6a, the GTV dose distribution using the homo-boron method demonstrates that dose variations are aligned with changes in exponential neutron distribution. Conversely, Fig. 6b adopts a non-uniform boron distribution, with the dose hot-spot and cold-spot regions corresponding to the boron distribution as visualized in the PET image presented in Fig. 1b. Setting aside the RBE and CBE of weighting factors, Fig. 7 provides insight into the physical dose rate variation utilizing both the aforementioned methods. Owing to the substantial contribution of boron-10 to the tumor dose, the degree of physical dose variation primarily aligns with the patterns observed in Fig. 6.

**Figure 8:**
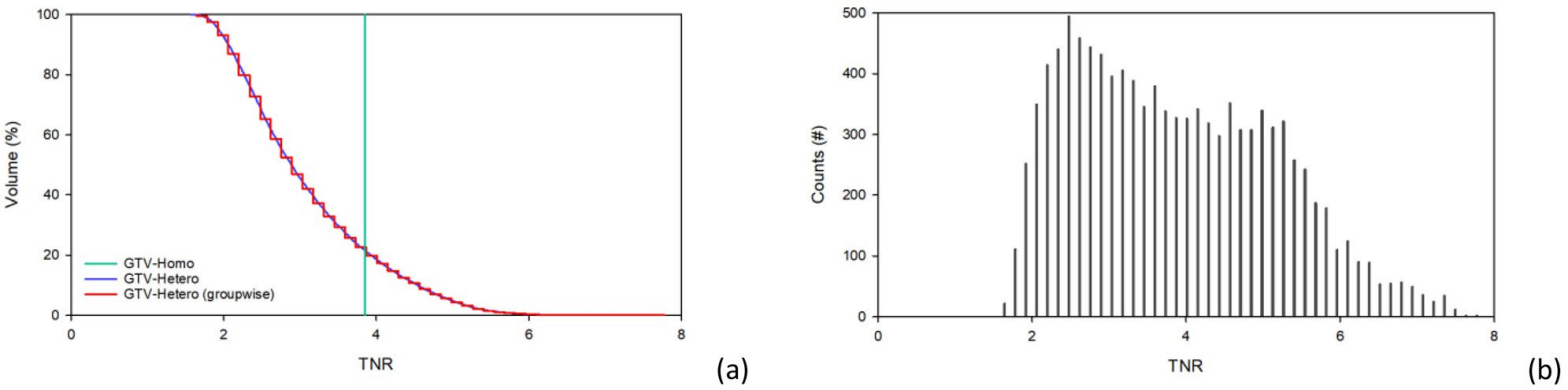
(**a**) TNR-volume histogram, (**b**) TNR-counts bar chart for GTV for Case 3.

**Figure 9:**
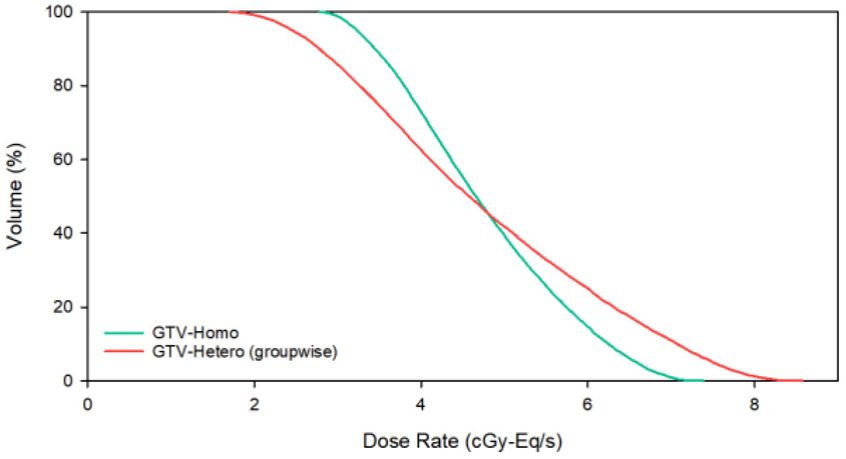
DVH of GTV with homo-boron and hetero-boron methods for Case 3.

In Case 3, the average TNR of the GTV is 3.85, ranging from 1.57 to 7.70, and more than 82% of the GTV volume is higher than 2.5. Figure 8 shows the TNR-volume histogram and the TNR-counts bar chart. Table 7 shows the same ${\dot{D}}_{mean}$ is obtained in the GTV using the homo-boron and hetero-boron methods. The difference between the ${\dot{D}}_{min}$ and ${\dot{D}}_{80}$ obtained using the two methods are 38.3% and 14.1%, respectively. The TNR at GTV ${\dot{D}}_{min}$ is 2.05 using the hetero-boron method, which is 46.8% lower than that obtained using the homo-boron method, and the corresponding ${\dot{D}}_{B}$ is 1.480 cGy-Eq/s in Table 8. The TNR at GTV ${\dot{D}}_{min}$ is 3.85 using the homo-boron method, and the corresponding ${\dot{D}}_{B}$ is 2.614 cGy-Eq/s, which is an overestimate of 43.4%. The green line in Fig. 9 is the result obtained using the homo-boron method, where the dose distribution ranges from 2.796 to 7.349 cGy-Eq/s shown in Table 7. The red line is the result obtained using the hetero-boron method, where the dose distribution ranges from 1.724 to 8.526 cGy-Eq/s.

**Table 7:** Dose rate results for Case 3 obtained using the homo-boron and hetero-boron methods.

ROI	Method	${\dot{D}}_{max}$(cGy-Eq/s)	${\dot{D}}_{min}$(cGy-Eq/s)	${\dot{D}}_{mean}$(cGy-Eq/s)	${\dot{D}}_{80}$(cGy-Eq/s)
GTV	Homo-boron	7.349	2.796	4.762	3.802
	Hetero-boron	8.526	1.724	4.762	3.265
	Difference	16.0%	− 38.3%	0.0%	− 14.1%

**Table 8:** Components of the dose rate at the tumor ${\dot{D}}_{max}$ and ${\dot{D}}_{min}$ dose category for Case 3 obtained using the homo-boron and hetero-boron methods.

Dose Category	Method	TNR	${\dot{D}}_{B}$(cGy-Eq/s)	${\dot{D}}_{N}$(cGy-Eq/s)	${\dot{D}}_{P}$(cGy-Eq/s)
GTV ${\dot{D}}_{max}$	Homo-boron	3.85	7.001 (95.3%)	0.115 (1.6%)	0.232 (3.2%)
	Hetero-boron	5.38	7.921 (96.1%)	0.102 (1.2%)	0.219 (2.7%)
	Difference	39.7%	13.1%	− 11.3%	− 5.6%
GTV ${\dot{D}}_{min}$	Homo-boron	3.85	2.614 (93.5%)	0.038 (1.4%)	0.143 (5.1%)
	Hetero-boron	2.05	1.480 (88.8%)	0.037 (2.2%)	0.150 (9.0%)
	Difference	− 46.8%	− 43.4%	− 2.6%	4.9%

**Figure 10:**
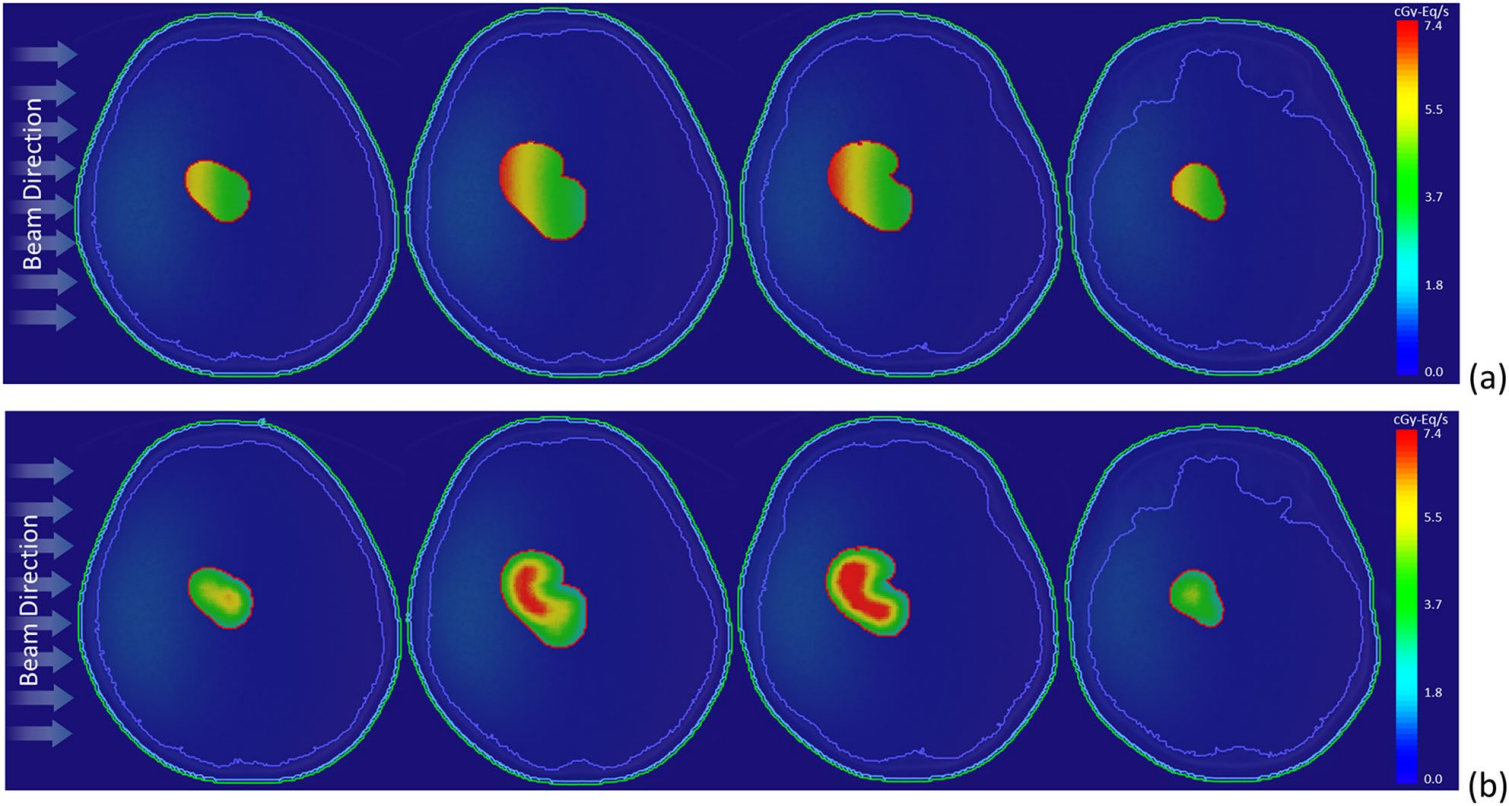
Bioequivalent dose maps of (**a**) homo-boron method, and (**b**) hetero-boron method for Case 3.

**Figure 11:**
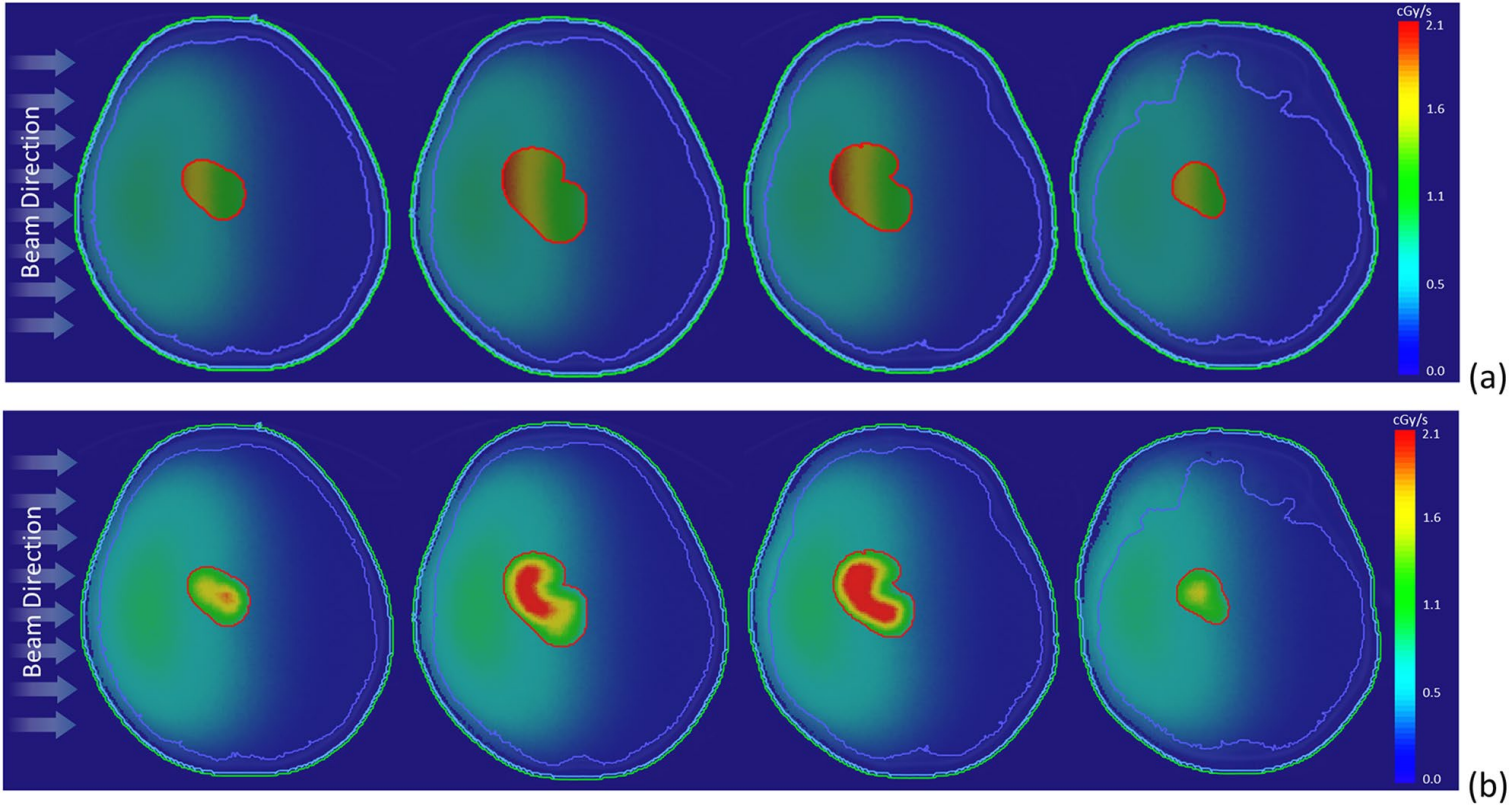
Physical dose maps of (**a**) homo-boron method, and (**b**) hetero-boron method for Case 3.

Figure 10 presents the bioequivalent dose maps, while Fig. 11 showcases the physical dose maps, both employing homo-boron and hetero-boron methods. The comparison between these figures highlights a distinct difference in the dose map, depending on the assumptions made regarding boron distribution. This divergence can be attributed to two distinct approaches: the assumption of a uniform boron distribution with a fixed TNR and the employment of a non-uniform boron distribution informed by PET imaging. When the homo-boron method is utilized, the dose hot-spot within the tumor is invariably localized in alignment with the direction of the incident beam. Conversely, regions further removed from the incident beam side manifest as dose cold-spots. It is important to recognize, however, that the actual distribution of boron exerts an influence on both the neutron and dose distributions, reflecting the complex interplay between these variables in the context of BNCT.

## Discussion

Traditionally, the nuclear medicine department utilizes threshold setting to ascertain the SUV_mean_ of both the tumor area and normal brain tissue with discernible drug accumulation in PET images, thereby determining the TNR. Concurrently, the radiation oncology department relies on CT images to define the GTV, which may surpass the delineation achieved through PET imaging. When the GTV exceeds the ROI defined by PET, employing a uniform TNR model—wherein a fixed, predetermined TNR represents the boron distribution within the GTV—results in an overestimation of the mean dose rate ${\dot{D}}_{mean}$ of the GTV. If the GTV in CT images corresponds to the tumor area in PET images, comparable ${\dot{D}}_{mean}$ values for the GTV can be obtained using both homogeneous and heterogeneous boron distributions, given their similar total neutron fluxes within the GTV. The intratumoral boron dose is fundamentally governed by the product of the spatial distribution of boron-10 atoms and thermal neutrons.

Despite similarities in the GTV ${\dot{D}}_{mean}$ between the homo-boron and hetero-boron methods, disparities exist between the dose hot-spots and cold-spots within the tumor due to variations in boron distribution. These disparities may even influence dose outcomes in critical surrounding organs. The methodology introduced in this study to quantify non-uniform boron distribution, based on ^18^F-BPA PET images, offers a more accurate dose distribution representation compared to a uniform boron distribution model. Discrepancies in voxel size between PET and CT images may induce a partial volume effect, becoming more pronounced as the target volume diminishes.

The study's findings reveal that employing the homo-boron method to define the GTV dose distribution leads to overestimation of the minimum dose rate ${\dot{D}}_{min}$ and ${\dot{D}}_{80}$, particularly in regions where the GTV may encompass a very low TNR portion, potentially even less than 1. This overestimation could obscure cold-spots within the GTV, where insufficient boron concentration may contribute to future recurrence or disease progression. Addressing potential cold-spots may require complementary radiation therapy modalities, such as photon therapy, proton therapy, or heavy ion therapy, to enhance tumor control.

In the present study, PET/CT imaging is utilized, with the two modalities being co-registered. Despite this integration, the actual crafting of treatment plans relies predominantly on either CT simulator images or MRI images. It is crucial to underscore that when the imaging data designated for treatment planning and the SUV metadata derived from PET scans originate from disparate equipment or temporal points, an alignment and fusion of these datasets become essential. However, this alignment process is not without its challenges; it may harbor varying degrees of discrepancies. As a result, our approach mandates a judicious evaluation of any potential errors and inaccuracies that could be introduced during the image fusion process.

In summary, this work sheds light on the impact of heterogeneous boron distribution on dose distribution within the GTV and offers valuable guidance on processing TNR based on PET images, contributing to more informed therapeutic decisions in BNCT.

## Future work

This study lays the groundwork for quantifying heterogeneous boron distribution utilizing PET images. However, several aspects warrant further investigation:

1. Optimization of TNR Groups: Determining the optimal total number of TNR groups to be employed in the groupwise approach requires comprehensive analysis and experimentation.
2. Diverse Case Analysis: Future research must encompass the analysis of a more extensive and varied set of cases, encompassing diverse target sizes and positions. Such analysis will contribute to the robustness of the method, ensuring its applicability across different scenarios in BNCT.

By addressing these aspects, subsequent research can build on the present study's findings, enhancing the precision and effectiveness of boron distribution quantification within the context of BNCT.

## Data Availability

The datasets used and/or analyzed during the current study available from the corresponding author on reasonable request.
